# Differential *Helicobacter pylori* Plasticity in the Gastric Niche of Subjects at Increased Gastric Cancer Risk

**DOI:** 10.3390/pathogens8020065

**Published:** 2019-05-18

**Authors:** Mariateresa Casarotto, Chiara Pratesi, Ettore Bidoli, Stefania Maiero, Raffaella Magris, Agostino Steffan, Giancarlo Basaglia, Vincenzo Canzonieri, Valli De Re, Renato Cannizzaro, Stefania Zanussi

**Affiliations:** 1 Immunopathology and Cancer Biomarkers Unit, Centro di Riferimento Oncologico di Aviano (CRO) IRCCS, 33081 Aviano, Italy; mtcasarotto@cro.it (M.C.); chiara.pratesi@aas5.sanita.fvg.it (C.P.); asteffan@cro.it (A.S.); vdere@cro.it (V.D.R.); 2 Cancer Epidemiology Unit, Centro di Riferimento Oncologico di Aviano (CRO) IRCCS, 33081 Aviano, Italy; bidolie@cro.it; 3 Oncological Gastroenterology Unit, Centro di Riferimento Oncologico di Aviano (CRO) IRCCS, 33081 Aviano, Italy; smaiero@cro.it (S.M.); raffaella.magris@cro.it (R.M.); rcannizzaro@cro.it (R.C.); 4 Microbiology and Virology Unit, Azienda per l’Assistenza Sanitaria n. 5 Friuli Occidentale, 33170 Pordenone, Italy; giancarlo.basaglia@aas5.sanita.fvg.it; 5 Pathology Unit, Centro di Riferimento Oncologico di Aviano (CRO) IRCCS, 33081 Aviano, Italy; vcanzonieri@cro.it

**Keywords:** gastric cancer, first degree relatives of gastric cancer patients, autoimmune gastritis, *Helicobacter pylori*, virulence factors, genetic heterogeneity

## Abstract

*Helicobacter pylori* (*H. pylori*) represents an independent risk factor for Gastric Cancer (GC). First Degree Relatives (FDR) of GC subjects and Autoimmune Gastritis (AG) patients are both at increased risk for GC. *H. pylori* genetic heterogeneity within the gastric niche of FDR and AG individuals has been little explored. To understand whether they exploit an increased *H. pylori* stability and virulence, 14 AG, 25 FDR, 39 GC and 13 dyspeptic patients (D) were investigated by a cultural PCR-based approach characterizing single colonies-forming-units. We chose three loci within the Cytotoxin-associated gene-A Pathogenicity Island (CagPAI) (*cagA*,*cagE*,*virB11*), *vacA, homA* and *homB* as markers of virulence with reported association to GC. Inflammatory/precancerous lesions were staged according to Sydney System. When compared to D, FDR, similarly to GC patients, were associated to higher atrophy (OR = 6.29; 95% CI:1.23–31.96 in FDR; OR = 7.50; 95% CI:1.67–33.72 in GC) and a lower frequency of mixed infections (OR = 0.16; 95% CI:0.03–0.81 in FDR; OR = 0.10; 95% CI:0.02–0.48 in GC). FDR presented also an increased neutrophil infiltration (OR = 7.19; 95% CI:1.16–44.65). Both FDR and GC carried a higher proportion of CagPAI^+^*vacAs1i1mx^+^homB^+^* profiles (OR = 2.71; 95% CI: 1.66–4.41 and OR = 3.43; 95% CI: 2.16–5.44, respectively). Conversely, AG patients presented a lower frequency of subtypes carrying a stable CagPAI and *vacAs1i1mx*. These results underline different *H. pylori* plasticity in FDR and AG individuals, and thus, a different host-bacterium interaction capacity that should be considered in the context of eradication therapies.

## 1. Introduction

*H. pylori* has presumably co-evolved with humans for at least 50,000 years to be transmitted from person to person and become a commensal of the stomach [1,2]. An equilibrium between *H. pylori* and host responses allows microbial persistence resulting in an increased risk of gastric neoplasia. *H. pylori* has been classified as a class I human carcinogen by the International Agency for Research on Cancer working group for its association, in particular, with non-cardia gastric cancer (GC) and mucosa-associated lymphoid tissue (MALT) lymphoma [3]. Since then, *H. pylori* infection has been shown to be the primary cause of gastric neoplasms [4], although its effects are multi-factorial. The mechanisms by which *H. pylori* may express its pathogenetic potential is related to bacterial structure and induced chronic inflammation, that triggers chronic active gastritis and development of GC lesions according the currently accepted model of precancerous Correa’s cascade (in order, non-atrophic gastritis, multifocal atrophic gastritis, intestinal metaplasia, dysplasia and, finally, cancer) [5].

Histopathological changes in the gastric mucosa can be associated with *H. pylori* fitness adaptation through multiple and subtle genetic events which allow its persistence in the microenvironment [6]. As a consequence, each host is colonized by a multitude of genetically closely related microorganisms, similar to quasispecies, which interfere with signaling pathways influencing host cell growth and death [7,8,9]. Several studies suggested a functional relation of particular combinations of genes and proteins, determining certain traits of *H. pylori* and specific pre-cancerous or pathological conditions [10,11,12,13,14,15]. In particular, the composition of the Cytotoxin-associated gene A Pathogenicity Island (CagPAI) modulates bacterial motility, survival, production of proinflammatory cytokines and antimicrobial susceptibility [16,17,18,19,20]. It has been highlighted that a single *H. pylori* strain may include variable proportions of subtypes with different CagPAI genotypes [21,22]. Deletions of CagPAI genes were more frequently detected among individuals with metaplasia and atrophic gastritis than non-atrophic gastritis or duodenal ulcers [23,24]. Another *H. pylori* virulence factor is the vacuolating toxin A (VacA) [25,26]. Different VacA isoforms are generated through the combination of three polymorphic regions, namely the signal (s), the intermediate (i) and the middle (m) regions, which affect the anion-selective channels formation, the vacuolating activity and the binding to different cell surface receptors, respectively [27,28,29,30]. The outer membrane protein (OMP) family includes surface molecules that are involved in *H. pylori* adherence and in the induction of a robust inflammatory response. Among OMP genes, *homA* and *homB* are poorly studied [31]. *HomB* had been associated with GC in USA, Colombia and Iran and a lower frequency of *homA* had been evidenced in patients with GC compared to those with chronic gastritis [32,33]. However, very few studies have examined the association between *hom* genes and GC in European countries [34].

Several studies have shown that long term colonization by specific *H. pylori* strains and the outcome of the infection are strictly dependent on interactions between *H. pylori* and the host genetic factors [25,35,36,37,38]. Subjects with a family history of GC or affected by autoimmune gastritis (AG) displayed a 1.5–3.0 fold higher risk to develop GC when compared to the general population [39,40,41] and in these individuals *H. pylori* is a recognized causative agent of gastritis. However, the importance of *H. pylori* virulence factors, along with conditions such as being First Degree Relatives (FDR) or having AG, which could increase the risk of GC development, has been little explored in non-endemic areas [42,43,44,45,46,47].

The aim of the present study is to understand whether an increased GC risk in populations, such as FDR and AG, could be associated with an intrinsic *H. pylori* high virulence. The results will increase the knowledge on *H. pylori* pathogenesis and have implications in guiding the choice of eradication in these patients. For this purpose, we dissected *H. pylori* strain heterogeneity in the gastric niche of FDR and AG individuals by using a focused genetic analysis on the most representative inflammation-related *H. pylori* virulence factors: *virB11*, *cagE* and *cagA*, located in the CagPAI, *homA* and *homB* genes, and *vacA s*, *m* and *i* regions. We applied virulent gene profiling on single bacterial isolates from primary plates obtained from the biopsies of each single patient (at least 10 colony-forming-units (CFU) per patient). Results could highlight possible virulence factors associated with predisposition to GC development in specific populations at risk for GC.

## 2. Results

### 2.1. Patients Characteristics

Patients were selected from a larger population performing endoscopy at the Oncological Gastroenterology Division, Centro di Riferimento Oncologico (CRO), Aviano (Italy) and submitted to *H. pylori* infection diagnostic workup. Inclusion criteria and strategies for variable clustering were defined in the Materials and Methods section. Table 1 shows the demographic characteristics of the subjects and the Sydney classification of gastric lesions related to the site of *H. pylori* isolation. When compared to dyspeptic patients (D), no statistically significant difference in the distribution of the patients by age and sex was observed. FDR were associated with a significantly higher gastric neutrophil activity (OR = 7.19; 95% CI: 1.16–44.65, *p =* 0.03) and glandular atrophy (OR = 6.29; 95% CI: 1.23–31.96, *p =* 0.03). This last characteristic was shared with GC patients (OR = 7.50; 95% CI: 1.67–33.72, *p =* 0.009), while the association of AG with atrophy was nearly significant (OR = 5.14; 95% CI: 0.81–32.77, *p =* 0.08). No statistically significant difference in intestinal metaplasia was evidenced by all the groups, while the degree of chronic inflammation and *H. pylori* density were similar in FDR and slightly lower in GC and AG when compared to D group.

### 2.2. H. pylori Putative Virulent Gene Load in the Gastric Niche

The *H. pylori* gastric niche was globally analyzed in each subject pooling the subtypes positive for the studied virulent factors. The concomitant presence of *cagA*, *cagE* and *virB11* was considered as an indicator of CagPAI stability. *VacA* haplotypes were clustered considering *s1i1mx* with a highest vacuolization property than *sxi2m2* haplotype. The presence of *homB* haplotype, which putatively confers to *H. pylori* higher level pathogenicity than *homA*, was also evaluated.

GC patients showed a higher risk than D to carry a stable CagPAI (OR = 19.20; 95% CI: 3.79–97.36, *p =* 0.0004), and a nearly significant association to the most virulent forms of the *vacA* gene (OR = 3.44; 95% CI: 0.83-14.17, *p =* 0.09) (Table 2). No difference in *homB* gene distribution was observed. A lower presence of *cagA* gene was registered in the *H. pylori* niche from AG subjects in comparison to D (OR = 0.17; 95% CI: 0.03–0.90, *p =* 0.04). Overall, AG patients showed a tendency to have a lower association with all the analyzed highly virulent factors. *VirB11* was the most conserved CagPAI gene in all the tested populations. We then evaluated the prevalence of *H. pylori* mixed infections defined as the presence in a patient of at least one subtype different from the others for at least one virulence factor. We showed that mixed infections were less frequent in GC (OR = 0.10; 95% CI: 0.02–0.48, *p =* 0.004) and FDR groups (OR = 0.16; 95% CI: 0.03–0.81, *p =* 0.03) compared to D, whereas no statistically significant difference was observed for AG.

### 2.3. Association Between H. pylori Virulence Factors within H. pylori Subtypes

**Figure 1:**
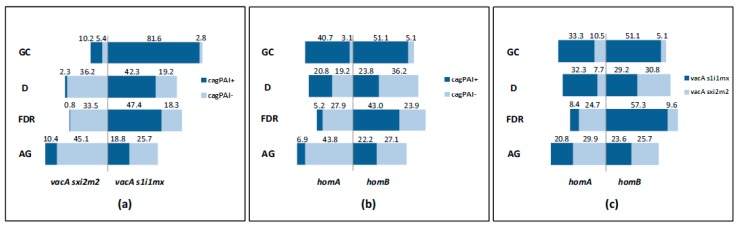
Schematic representation of the distribution of *H. pylori* subtypes by study group. (**a**) Frequency of *H. pylori* subtypes carrying a stable/unstable CagPAI associated to *vacA* variants; (**b**) frequency of *H. pylori* subtypes carrying a stable/unstable CagPAI associated to *hom* haplotypes; (**c**) frequency of *H. pylori* subtypes harboring the simultaneous presence of *hom* and *vacA* haplotypes. CagPAI^+^, subtypes with stable CagPAI (positive for *virB11*, *cagE* and *cagA*); CagPAI^-^, subtypes with at least one deletion for *virB11*, *cagE* or *cagA*.

CagPAI-positive *H. pylori* strains are most likely to carry the highly toxic forms of *vacA* [48]. Thus, we next analyzed whether there was any association among *vacA* haplotypes and a stable CagPAI in the subtypes, and we compared their frequencies within groups. An association between *vacA s1i1mx* forms and a stable CagPAI was shown in all the groups (OR = 15.18; 95% CI: 6.82-33.78, *p <* 0.0001 in GC; OR = 34.47; 95% CI: 9.78-121.42, *p <* 0.001 in D; OR = 108.65; 95% CI: 25.66-459.99, *p <* 0.0001 in FDR; OR = 3.16; 95% CI: 1.50-6.69, *p =* 0.003 in AG) (Table 3). The frequency of the subtypes with the coexistence of these virulence factors was particularly high in GC (81.6% of the subtypes) (Figure 1a). In this group, the less virulent *vacA sxi2m2* subtypes were also mainly associated to the presence of a stable CagPAI, but this genotype was present only in a minority of cases (10.2% of the subtypes) (Figure 1a). Subtypes carrying *vacA s1i1mx* and stable CagPAI were the less represented within the AG group (18.8% of the subtypes) (Figure 1a), and in comparison to D (OR = 0.33; 95% CI: 0.17–0.66, *p =* 0.0016).

*HomB* was found to coexist with the most virulent genotypes [31]; hence, we further assessed whether *hom* haplotypes were associated to the presence of CagPAI or *vacA* haplotypes. The frequency of *homB* haplotype was slightly higher in the GC group if compared to *homA* (56.2% versus 43.8% of the subtypes) (Figure 1b,c). However, while the subtypes with a stable CagPAI were equally associated with *homB* and *A* genes (51.1% and 40.7% of the subtypes) (Figure 1b), the virulent form of *vacA* showed a significant high risk of being linked with *homB* (OR = 3.14; 95% CI: 1.76–5.60, *p =* 0.0001) (Table 3). In the D group subjects, the distribution of the subtypes carrying a stable CagPAI was similar independently of *hom* status (23.8% in *homB*, 20.8% in *homA*) (Figure 1b); a low association between *homB* and *vacA s1i1mx* was found (OR = 0.23; 95% CI: 0.10–0.51, *p =* 0.0004) (Table 3).

FDR showed a high proportion of subtypes displaying *homB* associated to the presence of a stable CagPAI (43.0% of the subtypes) (Figure 1b) (OR = 9.70; 95% CI: 5.00–18.96, *p <* 0.0001) (Table 3) or *vacA s1i1mx* (57.3% of the subtypes) (Figure 1c) (OR = 17.71; CI: 9.18–34.17, *p <* 0.0001) (Table 3). These associations were statistically significant also in comparison to D (*homB* and stable CagPAI compresence: OR = 2.73; 95% CI: 1.57-4.74, *p =* 0.0004; *homB* and *vacA s1i1mx* compresence: OR = 7.58; 95% CI: 4.02–14.29, *p <* 0.0001). Thus, subtypes owning all the three virulence factors (stable CagPAI, *vacAs1i1mx* and *homB*) were significantly associated with FDR (OR = 2.71, 95% CI: 1.66–4.41, *p =* 0.0001) as demonstrated by GC (OR = 3.43, 95% CI: 2.16-5.54, *p <* 0.0001) (Table 4). Although with a low statistical significance, the highly virulent profile appeared to be more prevalent in FDR and GC patients with intestinal metaplasia than in those without (OR = 9.33; 95% CI: 0.85–101.96, *p =* 0.07 in FDR; 3.18; 95% CI: 0.86–11.79, *p =* 0.08 in GC).

Concerning AG, we found a similar proportion of subtypes harboring *homA* and *homB* genes within this group (50.7% and 49.3%, respectively) (Figure 1b,c). However, a high frequency of subtypes carrying *homA* associated with an unstable CagPAI was present within this group (43.8% of the subtypes) (Figure 1b) (OR = 5.17; 95% CI: 2.29–11.67, *p =* 0.0001) (Table 3). Although not significant, *H. pylori* subtypes from AG exploited a reduced risk to carry all the three virulence factors compared to D (OR = 0.84, 95% CI: 0.47–1.52, *p =* 0.57) (Table 4).

## 3. Discussion

It has been proposed that *H. pylori* infection and host factors play an important role in determining the clinical outcome of the infection [25,49]. Differences in *H. pylori* strains concerning specific virulent genes could be involved in the progression of gastric precancerous lesions to GC [10,11]. However, *H. pylori* strains exhibit a high degree of heterogeneity that helps its adaptation to and persistence in an evolving gastric environment [9,50]. To evaluate if populations with an increased GC risk could be associated with an intrinsic heightened virulence of the bacterium, molecular analyses of *H. pylori* isolates assessing the heterogeneity of the virulent gene profile were performed in AG and FDR subjects, and compared to those obtained from *H. pylori* subtypes isolated from D, and from GC as a positive control.

Consistent with data reported in the literature [49,51,52,53], GC patients included in this study showed an association with *H. pylori* strains harboring the CagPAI, which was accompanied by the presence of the highly virulent forms of the *vacA* gene, in accordance with previously reported data [54,55]. However, although some authors evidenced an association between GC and *H. pylori* strains positive for *homB* gene [32,33], our population-based analysis did not confirm this relation. This discrepancy could be explained by the different geographical origin of the patients from whom the strains had been isolated, and by the use of different preanalytical approaches [32,33,34,56]. Indeed, when we considered the *H. pylori* heterogeneity by the characterization of several subtypes shaping the gastric niche, we found that GC patients appear to host a high proportion of *homB* associated with the virulent form of *vacA*, and have a significant risk to carry subtypes containing all the three virulence factors.

In this study, *H. pylori* isolates from FDR mainly carried the highest virulent *vacA s1i1mx* genotype combined with a stable CagPAI. A similar *vacA* haplotype distribution was also evidenced in the D group, consistent with previous results obtained in studies conducted on similar cohorts [42,43]. For the first time, we assessed the presence of *hom* genes in FDR and AG populations. We showed a high frequency of subtypes carrying the *homB* haplotype associated both to a stable CagPAI and to *vacA s1i1mx,* specifically in FDR. In the latter group, similarly to GC patients, a high frequency of the genotype including all the three virulence factors was evidenced. It has been reported that *homB* was more frequent in *H. pylori* strains carrying *cagA* gene and the most virulent forms of *vacA* [31,32]; moreover, in the presence of the CagPAI, *homB* was found to promote a considerable proinflammatory response in vitro [31]. These observations suggest that *homB* might have a role in the development of a more severe clinical outcome of the infection in subjects such as FDR, who present a higher risk for GC than the general population. In addition, we interestingly found a lower prevalence of mixed infection in FDR than in D. It is well known that mixed infections determine the availability of exogenous DNA, which allows bacterial genome diversification and adaptation to an unfavorable environment [57]. Indeed, a higher frequency of recombination events during chronic infections have been reported in genes that influence bacterial adherence to epithelial cells and immune response [9,58]. It could be hypothesized that the reduced frequency of mixed infections in FDR could be the result of a selective pressure from the host, which promotes the emergence of *H. pylori* subtypes able to exacerbate the recruitment of immune cells at the site of infection in an attempt to reduce the bacterial survival, but actually reducing the possibilities of virulence attenuation. In accordance with this hypothesis, histological analyses in the present and other studies revealed a significant higher frequency of neutrophil infiltration (activity) and atrophy in the gastric mucosa of FDR when compared to that of D [44,59,60]. Moreover, IL-8 up-regulation and a more severe inflammatory reaction during *H. pylori* infection have been documented in FDR [61,62]. Our data, showing the predominance of more virulent *H. pylori* strains in FDR, reinforce the model involving a contribution of *H. pylori* in the progression of precancerous lesions towards GC in this population. The fact that any significant difference in *H. pylori* density was evidenced in FDR compared to D supports the importance of specific host and bacterial features rather than the quantity of *H. pylori* in determining the type of the response to the infection.

Then, we analyzed patients with antiparietal cell autoantibodies. It is reported that this condition precedes the onset of severe forms of autoimmune gastritis that expose the patients to the risk of non-cardia gastric adenocarcinoma [63,64]. Consistent with data previously reported, in our AG patients, vital *H. pylori* was isolated from a minority of cases (21%) [65,66,67,68]. The low recovery of *H. pylori* from AG subjects could suggest the presence of low amounts of *H. pylori* strains in their gastric niche. Indeed, we found a lower *H. pylori* density (Sydney 2-3) in AG (7%) than D (31%, Table 1). Low *H. pylori* amounts could be related to low fitness, which in turn could be dependent on changes in gastric relative abundance of other bacterial species that could compete for the vital space within the niche [69,70], participating in the gastric pathogenesis and in the exchange of resistance determinants [71,72]. It is worth noting that, in a subset of AG patients, we found a high proportion of individuals carrying a heterogeneous bacterial flora [73]. Herein, a high prevalence of Streptococcaceae was evidenced by a semiquantitative cultural method (data not shown), in agreement with recent studies conducted on AG series [69]. *H. pylori* isolated from AG showed a significant reduced risk to carry the *cagA* gene and a reduced frequency of all the evaluated virulence factors; as the chance to develop GC is higher when virulence bacterial factors are present, our findings are in agreement with the observation that only a small percentage of AG patients with active *H. pylori* infection is at higher risk to develop GC [40]. In these AG patients, we found that subtypes carrying unstable CagPAI associated with *homA* or *vacA sxi2m2* haplotypes were the most represented. These results confirm that *homA* is linked with strains lacking some important *H. pylori* virulence genes [31,32], which, on the other hand, suggest an adaptation of the bacterium to the particular gastric environment produced during the chronic autoimmune disorder [2,74,75]. The residence of less virulent subtypes probably owning a higher fitness in the gastric niche and a different interaction capacity with the host is supported by a previous proteomic study revealing that *H. pylori* strains isolated from AG subjects have a tendency to express proteins involved in survival and bacterial protection from the gastric environment, rather than molecules involved in biosynthetic pathways and in invasion of the gastric mucosa [14]. Immune response over the long course of *H. pylori* colonization might play a role in selecting *H. pylori* subtypes which display target bacterial genes [37]. A role of chronic *H. pylori* infection in the development of AG has been previously proposed on the basis of a positive correlation between parietal auto-antibodies and antibodies specific for *H. pylori* antigens in the majority of AG patients, a phenomenon explained by molecular mimicry [76,77]. Homologies between self and *H. pylori* proteins, such as CagA, have been documented [78,79,80,81,82]. It is conceivable that, in inflammatory chronic diseases with autoimmune components and *H. pylori* infection, the equilibrium between *H. pylori* and host immune mechanisms might contribute to the removal of the *H. pylori* more virulent strains carrying CagA, as was found in our AG series [83,84].

The possible association of *H. pylori* with AG, related to the antigenicity of specific virulence traits involved in the initiation of the disease and in the modulation of *H. pylori* pathogenicity, does not exclude that the bacterium might contribute to support the functional loss of the stomach through the persistence of its virulence factors. By dissecting individual histological features and associated bacterial characteristics, we found a predominance of subtypes with stable CagPAI in 4 out of 14 AG patients, who had a higher median age than those hosting subtypes with CagPAI deletions. Interestingly, three of these four patients had a moderate corpus atrophy (Sydney 2). Whole genome sequencing studies evidenced a temporal and spatial evolution of *H. pylori* genome, with gain and loss of multiple virulence and resistance genes [85,86,87]. However, CagPAI appears to be relatively stable during disease progression to gastric carcinoma [86]. This highlights the possibility that AG patients could also be associated with a persistently virulent *H. pylori*. Hence, eradication therapy may be indicated in a subset of these patients, especially in the presence of signs of atrophy or old age. However, it must be kept in mind that the intactness or deletions of specific virulence factors, such as CagPAI and *cagA,* or the presence of less virulent forms of *vacA,* could be associated with phenotypic resistance to antibiotics [19,88,89,90,91] and high risk of eradication failure [92]. These data imply that, in the presence of diversity within single *H. pylori* strains, antibiotics therapies could select resistant subtypes and, in addition, they stimulate research on feasible methods to assess the presence of resistance within heterogeneous populations.

The present study has some limitations and merits. First, the number of the patients could be considered low, and made possible only univariate analyses. However, for the first time the presence of some *H. pylori* virulence genes has been assessed in non-endemic populations at risk to develop GC, such as FDR and AG, thoroughly analyzing 10–12 single colonies isolated from biopsies of single patients. This could not be representative of the entire heterogeneity of the *H. pylori* gastric niche, although this number was higher in comparison to that investigated in other studies with similar culture-based approaches [93,94]. An implementation of the study could derive from investigations on biopsies collected on a greater number of paired antrum-corpus samples, given that our study was basically conducted on biopsies isolated from the preferential niche of *H. pylori*, the antrum region. Second, if compared to more advanced molecular techniques which explore the entire *H. pylori* genome [58], the method we used allowed to examine a limited set of *H. pylori* virulence factors. However, the sensitivity of PCR-based virulent profiling of multiple *H. pylori* subtypes is higher if compared with other methods, such as RFLP and RAPD PCR [21,95]. In the future, the complete genome sequence data could deepen this point, providing information about still unknown bacterial genes [86]. Finally, our study produced a collection of well-characterized bacterial isolates useful to dissect specific pathogenetic mechanisms of *H. pylori*.

Multicenter perspective studies enrolling a high number of patients at early phases of gastric diseases and performed with high throughput molecular techniques avoiding time-consuming bacterial culture [96] could improve the knowledge of *H. pylori* patho-physiology and the management of individuals at risk of GC.

## 4. Materials and Methods 

### 4.1. Study Population

A total of 340 subjects who underwent gastrointestinal endoscopy at the Oncological Gastroenterology Division, Centro di Riferimento Oncologico (CRO), Aviano (Italy) were submitted to *H. pylori* infection diagnostic workup. All the patients were subjected to an accurate clinical interview to ascertain the presence of already diagnosed diseases and current or recent pharmacological or surgical treatments. Subjects were included in the study according to the following criteria: positive culture for *H. pylori,* absence of previous *H. pylori* eradication therapy, of continuous or occasional proton pump inhibitor or antibiotic cures in the last month, and of chronic nonsteroidal anti-inflammatory drug treatments. Patients affected by gastritis and having auto-antibodies against parietal cells were defined as AG. Antiparietal cell antibody levels were estimated by means of indirect immunofluorescence with a cutoff of ≥1/80 (Euroimmun, Lubeck, Germany). Subjects with one or more cases of GC among their first-degree relatives were defined as FDR. Dyspeptic subjects without familiarity or autoimmunity (D) were included in the study as controls. In addition, confirmed GC cases were evaluated for the known association between *H. pylori* virulence factors and the pathology. Positive *H. pylori* cultures were obtained from 14 out of 67 AG (20.9%), 26 out of 105 FDR (24.8%), 42 out of 126 GC (33.3%) and 16 out of 42 D (38.1%). Seven subjects were subsequently excluded due to the loss of viability or bacteria contamination during culture steps. 39 high GC risk (14 AG, 25 FDR), 39 GC and 13 D were finally involved in the analysis. The study was approved by the Internal Review Board of CRO, Aviano (Italy) (IRB 14-2013). All study participants, or their legal guardian, provided informed written consent before enrollment. Ethical guidelines for research involving human subjects were respected.

### 4.2. Endoscopy and H. pylori Culture and Identification

Multiple biopsy specimens were taken for histological examination: two biopsies from the antrum, two from the corpus and two from the fundus. One antrum biopsy from all the patients, and one additional corpus biopsy from four FDR, four AG, eight D and 10 GC subjects were received in the laboratory and cultured for *H. pylori*. The specimens were obtained by using sterilized biopsy forceps, which were cleansed with a detergent, rinsed with sterile water after each examination. Biopsies were collected in a vial containing 1 mL of 0.9% NaCl solution and delivered to the laboratory within 2 hours. After subtle fragmentation, they were cultured in Pylori Selective Medium (Bio-Mérieux, Florence, Italy) following standard cultivation conditions for *H. pylori* [97]. Briefly, cultures were incubated at 37°C under sachet-generating microaerophilic athmosphere (Oxoid, Basingstoke, UK) for 3–5 days. If growth was not observable, incubation was prolonged for further 9–11 days and culture plates were controlled for growth twice/thrice weekly before considering them definitively negative. Concerning patients involved in this study, negative cultures were obtained from the antrum of two AG patients and from the corpus of two GC patients. The cultured bacteria were identified as *H. pylori* based on Gram-negative staining, curved or spiral shape, and positivity for oxidase, catalase and urease production.

In order to represent the possible genetic heterogeneity of *H. pylori* populations that colonize the gastric niche, 10 to 12 CFU were isolated from each single *H. pylori* positive selective primary plate, as previously described [21,24]. In brief, each CFU was spotted on Columbia Sheep Blood Agar (CA) (Kima, Padua, Italy), and then expanded on three CA plates. Each incubation was carried out under microaerophilic atmosphere at 37°C for a maximum of 2–3 days and four passages, to limit the appearance of *H. pylori* coccoid form. After confluent bacterial growth, two plates were used for bacterial DNA extraction and one for cryo-conservation (Microbank, Pro-Lab Diagnostics, Richmond Hill, Canada). A total of 915 *H. pylori* single colonies defining possibly subtypes were isolated from 91 patients.

### 4.3. Histological Study

For histological examination, biopsy specimens were fixed in buffered formalin 10%, embedded in paraffin and stained with hematoxylin and eosin by means of the modified Giemsa method for *H. pylori*. Available slides were retrieved retrospectively: 13 antrum and 11 corpus for D, 14 antrum and 14 corpus for AG, 25 antrum and 21 corpus for FDR and 39 antrum and 19 corpus for GC patients. A pathologist, who was unaware of endoscopic, microbiological or serological data, evaluated the inflammatory and precancerous parameters in accordance with the Sydney System classification [98], assigning a score from 0 to 3 (absent, mild, moderate, severe) to each of the following structural variables: activity (amount of neutrophilic infiltration), inflammation (amount of mononuclear-cell infiltration), atrophy (loss of glandular tissue), intestinal metaplasia and *H. pylori* density. The Sydney System’s parameters related to the profiled gastric regions were included in the analyses of patients’ characteristics.

### 4.4. Genomic DNA Extraction

Grown bacteria were collected from the CA plates and resuspended in 1 mL of 0.9% NaCl. Bacterial pellets were then obtained by centrifugation at 6797g for 6 minutes. Genomic DNA was extracted by QIAmp DNA Mini Kit (QIAgen, Hilden, Germany) following manufacturer’s instructions. NanoDrop 1000 Spectrophotometer (Thermo Scientific) was used for the calculation of DNA concentration. A fixed amount of DNA corresponding to 4 ng was used in each PCR reaction.

### 4.5. PCR-Based Virulent Gene Profiling

Each PCR reaction was performed in a final volume of 50 μL containing 10mM Tris-HCl, 50 mM KCl, 1.5 mM MgCl_2_, 0.2 mM dNTPs, 0.5 μM of each primer, 0.020 U/μL of AmpliTaq® DNA Polymerase (Applied Biosystems, Foster City, CA, USA). After validation of the results in double reactions with known samples, for the evaluation of *vacA* polymorphisms and *hom* genes, 0.015 U/μL per reaction of GoTaq® G2 DNA Polymerase was used (Promega, Madison, WI, USA).

In order to speed-up the genotyping procedure, a different thermal profile was applied to a multiplex PCR including *cagA* and *vacA s* and *m* regions. The test was reliable and provided results overlapping those obtained through the previous single PCR assays. The multiplex PCR reaction mix contained 1.5 mM MgCl_2_, 0.25 mM dNTPs, 18 pmol of each *vacA* primer, 28 pmol of *cagA* primers, 0.8 U/μL of GoTaq® G2 DNA Polymerase (Promega, Madison, WI, USA) in a final volume of 25 μL. Primer sequences, thermal PCR conditions and length of the amplified fragments are reported in Table 5.

CagPAI and *hom* PCR products were analyzed by 8% polyacrylamide gel electrophoresis, while *vacA s*, *m* and *i* PCR products were loaded in 2% agarose gel electrophoresis. After ethidium bromide staining, all the gels were visualized under a ultraviolet transilluminator (Gel Doc 2000, Biorad).

Specificity of the amplification was confirmed by Sanger sequencing after PCR product purification with Centricon-100 concentrator columns (Amicon, Beverly, Mass). Sequencing reactions were run on ABI Prism 3130XL automated DNA Sequencer (Applied Biosystems).

### 4.6. Definitions and Statistical Analysis

Parameters under study were dichotomized on the basis of their frequencies in the studied groups. The presence of activity, atrophy and intestinal metaplasia was defined when the Sydney score was equal or greater than 1. A stratification of the patients by the grade of inflammation and *H. pylori* density 0–1 versus 2–3 was made. Age was dichotomized considering the median age of the D controlgroup.

*CagA*, *cagE* and *virB11* within a subtype were studied as single genes and as a whole: the concomitant presence of *virB11* (located in the left half of the CagPAI), *cagA* and *cagE* (both located in the right half of the CagPAI) in a subtype was considered as an indicator of CagPAI stability (herein named stable CagPAI); conversely, an unstable CagPAI was defined by the deletion of at least one of the aforementioned CagPAI genes. *VacA* haplotypes were clustered based on the presence of *s*, *m*, *i* allelic heterogeneity, considering *s1i1mx* with a highest vacuolization property than *sxi2m2* haplotype, and where *x* indicated the alternative allele 1 or 2 [25,28]. 23 out of 915 total subtypes without *vacA* (2.51%) were grouped with *sxi2m2* positive isolates. 44 out of 915 total subtypes carrying both *homB* and *homA* (4.81%) were assembled within the strains keeping the *homB* haplotype, which putatively confers higher level pathogenicity than *homA* [31]. The very few subtypes without *hom* (10/915, 1.09%) were grouped with strains carrying *homA*.

Since antrum is the preferential niche of *H. pylori* [101], when identical bacterial profiles from paired antrum corpus biopsy samples were obtained, those related to only the antrum were included in the statistical analyses (three in FDR, two in AG, six in D, eight in GC). In three cases (one in FDR and two in D) a difference in genotypes by topography was evidenced, nonetheless the profile related to antrum region was considered. Since no significant differences were shown in the distribution of the Sydney parameters between matched antrum and corpus samples (Supplementary Table S1), corpus-related *H. pylori* profiles of the two AG cases with negative biopsy culture from the antrum were included.

In order to evaluate the *H. pylori* putative virulent gene load in the gastric niche of the studied subjects, based on the frequency of the subtypes carrying virulent genetic traits in each group, an individual was *a priori* considered owner of a highly virulent *H. pylori* load in the niche when a number of subtypes ≥9 was positive for the virulent factors analyzed. A mixed infection in a patient was defined based on the presence of at least one subtype different from the others for at least one virulence factor.

Univariate Odds ratios (OR) and their corresponding 95% confidence intervals (CI) were computed to estimate the differences in the distribution of the patients by demographic, histological and virulent profiles and to assess virulence factor associations in the subtypes within and among the groups under study. Two-tailed *p*-value <0.05 was considered to indicate statistical significance. Analyses were performed by the SAS System software, version 9.40 (SAS Institute Inc., Cary, NC, USA, 1999–2001).

## 5. Conclusions

Less virulent *H. pylori* subtypes were mainly isolated from AG patients, whereas highly virulent *H. pylori* profiles predominated in FDR, suggesting a selection exerted by the host in the gastric niche. These results underline that differences in *H. pylori* genome might play differential roles in gastric pathogenesis. Our observations strengthen the guidelines statements that recommend eradication of the *H. pylori* infection in FDR subjects [102]. Eradication therapy in *H. pylori* positive subjects at high risk of GC should be considered in relation to the complexity of the factors involved in the gastric tumor development, not least, to the alterations of the gastric microbiota of which *H. pylori* is part and within which it can compete for survival [103,104].

## Figures and Tables

**Table 1:** Demographic characteristics and Sydney System classification of the studied subjects.

	GC	AG	FDR	D ^†^
	N = 39	N = 14	N = 25	N = 13
**Females,** n (%)	19 (48.72)	11 (78.57)	15 (60.00)	9 (69.23)
OR (95% CI)	0.42 (0.11−1.60)	1.63 (0.29−9.26)	0.67 (0.16−2.77)	1
Z-statistic	1.27	0.55	0.56	
*p*-value	0.21	0.58	0.58	
**Age ≥ 56 years**, n (%)	29 (74.36)	5 (35.71)	8 (32.00)	7 (53.85)
OR (95% CI)	2.49 (0.67−9.18)	0.48 (0.10−2.23)	0.40 (0.10−1.60)	1
Z-statistic	1.37	0.94	1.29	
*p*-value	0.17	0.35	0.20	
**Activity ≥ 1**, n (%)	32 (82.05)	11 (78.57)	23 (92.00)	8 (61.54)
OR (95% CI)	2.86 (0.72−11.41)	2.29 (0.42−12.50)	7.19 (1.16−44.65)	1
Z-statistic	1.49	0.98	2.12	
*p*-value	0.14	0.34	0.03	
**Inflammation 2-3**, n (%)	20 (51.28)	6 (42.86)	19 (76.00)	10 (76.92)
OR (95% CI)	0.32 (0.08−1.33)	0.23 (0.04−1.19)	0.95 (0.20−4.63)	1
Z-statistic	1.57	1.75	0.06	
*p*-value	0.12	0.08	0.95	
**Atrophy ≥ 1**, n (%)	35 (89.74)	12 (85.71)	22 (88.00)	7 (53.85)
OR (95% CI)	7.50 (1.67−33.72)	5.14 (0.81−32.77)	6.29 (1.23−31.96)	1
Z-statistic	2.63	1.73	2.22	
*p*-value	0.009	0.08	0.03	
**Intestinal metaplasia ≥ 1**, n (%)	19 (48.72)	3 (21.43)	5 (20.00)	4 (30.77)
OR (95% CI)	2.14 (0.56−8.12)	0.61 (0.11−3.49)	0.56 (0.12−2.60)	1
Z-statistic	1.12	0.55	0.74	
*p*-value	0.26	0.58	0.46	
**HP density 2-3**, n (%)	5 (12.82)	1 (7.14)	10 (40.00)	4 (30.77)
OR (95% CI)	0.33 (0.07−1.49)	0.17 (0.02−1.82)	1.50 (0.36−6.23)	1
Z-statistic	1.44	1.46	0.56	
*p*-value	0.15	0.14	0.58	

GC, Gastric Cancer; AG, Autoimmune Gastritis; FDR, First Degree Relatives; D, Dyspeptic patients; ^†^, reference category; N, number of patients; OR, odds ratio; CI, confidence interval; HP, *Helicobacter pylori.*

**Table 2:** *H. pylori* putative virulent gene load in the gastric niche of the studied subjects.

	GC	AG	FDR	D ^†^
	N = 39	N = 14	N = 25	N = 13
**CFU with stable CagPAI ≥ 9**, n (%)	36 (92.31)	4 (28.57)	12 (48.00)	5 (38.46)
OR (95% CI)	19.20 (3.79−97.36)	0.64 (0.13−3.20)	1.48 (0.38−5.79)	1
Z-statistic	3.57	0.54	0.56	
*p*-value	0.0004	0.59	0.58	
CFU with positive *cagA* ≥ 9, n (%)	36 (92.31)	5 (35.71)	16 (64.00)	10 (76.92)
OR (95% CI)	3.60 (0.63−20.65)	0.17 (0.03−0.90)	0.53 (0.12−2.46)	1
Z-statistic	1.44	2.08	0.81	
*p*-value	0.15	0.04	0.42	
CFU with positive *cagE* ≥ 9, n (%)	36 (92.31)	4 (28.57)	13 (52.00)	5 (38.46)
OR (95% CI)	19.20 (3.79−97.36)	0.64 (0.13−3.20)	1.73 (0.44−6.79)	1
Z-statistic	3.57	0.54	0.79	
*p*-value	0.0004	0.59	0.43	
CFU with positive *virB11* ≥ 9, n (%)	37 (94.87)	6 (42.86)	17 (68.00)	8 (61.54)
OR (95% CI)	11.56 (1.89−70.59)	0.38 (0.08−1.86)	1.06 (0.25−4.60)	1
Z-statistic	2.65	0.97	0.40	
*p*-value	0.008	0.33	0.69	
**CFU with *vacA s1i1mx* ≥ 9**, n (%)	33 (84.62)	4 (28.57)	16 (64.00)	8 (61.54)
OR (95% CI)	3.44 (0.83−14.17)	0.25 (0.05−1.25)	1.11 (0.28−4.43)	1
Z-statistic	1.71	1.69	0.15	
*p*-value	0.09	0.09	0.88	
**CFU with *homB* ≥ 9**, n (%)	22 (56.41)	7 (50.00)	16 (64.00)	7 (53.85)
OR (95% CI)	1.11 (0.31−3.91)	0.86 (0.19−3.89)	1.52 (0.39−5.95)	1
Z-statistic	0.16	0.20	0.61	
*p*-value	0.87	0.84	0.54	
**Mixed infection**, n (%)	3 (7.69)	5 (35.71)	3 (12.00)	6 (46.15)
OR (95% CI)	0.10 (0.02−0.48)	0.65 (0.14−3.03)	0.16 (0.03−0.81)	1
Z-statistic	2.85	0.55	2.22	
*p*-value	0.004	0.58	0.03	

GC, Gastric Cancer; AG, Autoimmune Gastritis; FDR, First Degree Relatives; D, Dyspeptic patients; ^†^, reference category; N, number of patients; CFU, colony-forming-units; CagPAI, Cytotoxin associated gene-A Pathogenicity Island; OR, odds ratio.

**Table 3:** Association between CagPAI status and *vacA* polymorphisms or *hom* haplotypes, and between *hom* haplotypes and *vacA* polymorphisms within *H. pylori* CFU by group.

		CFU with CagPAI N (%)		OR (95% CI)	Z *	*p*-Value	CFU with *hom* haplotype N (%)		OR (95% CI)	Z *	*p*-Value
Group (CFU N.)	Haplotypes	Unstable	Stable				*A*	*B*			
GC (390)	*vacAsxi2m2*	21 (65.63)	40 (11.17)	1 ^†^			41 (23.98)	20 (9.13)	1 ^†^		
	*vacAs1i1mx*	11 (34.37)	318 (88.83)	15.18 (6.82–33.78)	6.66	<0.0001	130 (76.02)	199 (90.87)	3.14 (1.76−5.60)	3.88	0.0001
	*homA*	12 (37.50)	159 (44.41)	1 ^†^			n.a.	n.a.	n.a.		
	*homB*	20 (62.50)	199 (55.59)	0.75 (0.36-1.58)	0.753	0.45	n.a.	n.a.	n.a.		
D (130)	*vacAsxi2m2*	47 (65.28)	3 (5.17)	1 ^†^			10 (19.23)	40 (51.28)	1 ^†^		
	*vacAs1i1mx*	25 (34.72)	55 (94.83)	34.47 (9.78–121.42)	5.51	<0.001	42 (80.77)	38 (48.72)	0.23 (0.10−0.51)	3.55	0.0004
	*homA*	25 (34.72)	27 (46.55)	1 ^†^			n.a.	n.a.	n.a.		
	*homB*	47 (65.28)	31 (53.45)	0.61 (0.30–1.24)	1.37	0.17	n.a.	n.a.	n.a.		
FDR (251)	*vacAsxi2m2*	84 (64.62)	2 (1.65)	1 ^†^			62 (74.70)	24 (14.29)	1 ^†^		
	*vacAs1i1mx*	46 (35.38)	119 (98.35)	108.65 (25.66–459.99)	6.37	<0.0001	21 (25.30)	144 (85.71)	17.71 (9.18−34.17)	8.58	<0.0001
	*homA*	70 (53.85)	13 (10.74)	1 ^†^			n.a.	n.a.	n.a.		
	*homB*	60 (46.15)	108 (89.26)	9.70 (5.00–18.96)	6.64	<0.0001	n.a.	n.a.	n.a.		
AG (144)	*vacAsxi2m2*	65 (63.73)	15 (35.71)	1 ^†^			43 (58.90)	37 (52.11)	1 ^†^		
	*vacAs1i1mx*	37 (36.27)	27 (64.29)	3.16 (1.50–6.69)	3.01	0.003	30 (41.10)	34 (47.89)	1.32 (0.68−2.55)	0.82	0.41
	*homA*	63 (61.76)	10 (23.81)	1 ^†^			n.a.	n.a.	n.a.		
	*homB*	39 (38.24)	32 (76.19)	5.17 (2.29–11.67)	3.95	0.0001	n.a.	n.a.	n.a.		

CFU, colony-forming-units; CagPAI, Cytotoxin associated gene-A Pathogenicity Island; OR, odds ratio; CI, confidence interval; N, number of CFU; GC, Gastric Cancer; D, Dyspeptic patients; FDR, First Degree Relatives; AG, Autoimmune Gastritis; *, Z statistic; ^†^, reference category; n.a., not applicable.

**Table 4:** Subtypes carrying highly virulent profile (concomitant presence of stable CagPAI, *vacA s1i1mx* and *homB*) by group.

Group	Highly Virulent Profile N CFU (%)		OR (95% CI)	Z *	*p*-Value
	yes	no			
D	28 (21.54)	102 (78.46)	1 ^†^		
AG	27 (18.75)	117 (81.25)	0.84 (0.47−1.52)	0.58	0.57
FDR	107 (42.63)	144 (57.37)	2.71 (1.66−4.41)	4.01	0.0001
GC	189 (48.46)	201 (51.54)	3.43 (2.16−5.44)	5.21	<0.0001

N, number; CFU, colony-forming-units; OR, odds ratio; CI, confidence interval; D, Dyspeptic patients; AG, Autoimmune Gastritis; FDR, First Degree Relatives; GC, Gastric Cancer; *, Z statistic; ^†^, reference category.

**Table 5:** Primers and PCR conditions for the virulent gene profiling.

Gene	Coordinates †	Primer	Primer sequences (5′ → 3′)	Amplicons Length (bp)	Thermal Conditions	Ref.
***virB11***	516343–517335	virB11 (F) virB11 (R)	TTAAATCCTCTAAGGCATGCTAC GATATAAGTCGTTTTACCGCTTC	491	95°C, 3′; 50 x (94°C, 1′; 49°C, 45″; 72°C, 45″); 72°C, 7′	[21]
***cagE***	538897–541848	cagE (F) cagE (R)	TTGAAAACTTCAAGGATAGGATAGAGC GCCTAGCGTAATATCACCATTACCC	508	95°C, 3′; 50 x (94°C, 1′; 53°C, 45″; 72°C, 45″); 72°C, 7′	[21]
***cagA***	543605–547108	cagA (F) cagA (R)	ATAATGCTAAATTAGACAACTTGAGCGA AGAAACAAAAGCAATACGATCATTC	128	95°C, 3′; 50 x (94°C, 1′; 48°C, 45″; 72°C, 45″); 72°C, 7′	[21]
***vacA*** ***s1/s2***	900011–903877	s1/s2 (F) s1/s2 (R)	ATGGAAATACAACAAACACAC CTGCTTGAATGCGCCAAAC	259/286	95°C, 5′; 35 x (95°C, 20″; 52°C, 20″; 72°C, 40″); 72°C, 7′	[99] ^‡^
***vacA*** ***m1/m2***		m1/m2 (F) m1/m2 (R)	CAATCTGTCCAATCAAGCGAG GCGTCTAAATAATTCCAAGG	570/645		
***vacA i1***		i1-i2 (F) i1 (R)	GYTGGGAYTGGGGGAAYGCCG TTAATTTAACGCTGTTTGAAG	426	95°C, 5′; 35 x (95°C, 20″; 55°C, 20″; 72°C, 40″); 72°C, 7′	
***vacA i2***		i1-i2 (F) i2 (R)	GYTGGGAYTGGGGGAAYGCCG GATCAACGCTCTGATTTGA	432		
**Multiplex for** ***cagA***, ***vacA s/m***	543605–547108 900011–903877	cagA (F) cagA (R)	ATAATGCTAAATTAGACAACTTGAGCGA AGAAACAAAAGCAATACGATCATTC	128	94°C, 3′; 35 x (94°C, 1′; 55°C, 1′; 72°C, 1′); 72°C, 10′	[100]
		s1/s2 (F) s1/s2 (R)	ATGGAAATACAACAAACACAC CTGCTTGAATGCGCCAAAC	259/286		
		m1/m2 (F) m1/m2 (R)	CAATCTGTCCAATCAAGCGAG GCGTCTAAATAATTCCAAGG	570/645		
***homA/homB***	726428–728401/ 962682–964688	hom (F) hom (R)	AGAGGGTGTTTGAAACGCTCAATA GGTGAATTCTTCTGCGGTTTG	128/161	95°C, 5′; 35 x (95°C, 30″, 60°C, 30″, 72°C, 17″); 72°C, 7′	[34]

**†**, coordinates are referred to *H. pylori J99* strain genome; ‡, modified from the cited reference.

## Supplementary Materials

The following are available online at https://www.mdpi.com/2076-0817/8/2/65/s1, Table S1: Sydney classification of histological parameters in AG patients by gastric topography.

## References

[1] Blaser M.J., Atherton J.C. (2004). *Helicobacter pylori* persistence: Biology and disease. J. Clin. Investig..

[2] Atherton J.C., Blaser M.J. (2009). Coadaptation of *Helicobacter pylori* and humans: Ancient history, modern implications. J. Clin. Investig..

[3] IARC (1994). Infection with *Helicobacter pylori*. Monogr. Eval. Carcinog. Risks Hum..

[4] Parkin D.M. (2006). The global health burden of infection-associated cancers in the year **2002**. Int. J. Cancer.

[5] Correa P., Piazuelo M.B. (2012). The gastric precancerous cascade. J. Dig. Dis..

[6] Cover T.L., Blaser M.J. (2009). *Helicobacter pylori* in health and disease. Gastroenterology.

[7] Saberi S., Douraghi M., Azadmanesh K., Shokrgozar M.A., Zeraati H., Hosseini M.E., Mohagheghi M.A., Parsaeian M., Mohammadi M. (2012). A potential association between *Helicobacter pylori* CagA EPIYA and multimerization motifs with cytokeratin 18 cleavage rate during early apoptosis. Helicobacter.

[8] Greenfield L.K., Jones N.L. (2013). Modulation of autophagy by *Helicobacter pylori* and its role in gastric carcinogenesis. Trends Microbiol..

[9] Suerbaum S., Josenhans C. (2007). *Helicobacter pylori* evolution and phenotypic diversification in a changing host. Nat. Rev. Microbiol..

[10] Plummer M., van Doorn L.J., Franceschi S., Kleter B., Canzian F., Vivas J., Lopez G., Colin D., Muñoz N., Kato I. (2007). *Helicobacter pylori* cytotoxin-associated genotype and gastric precancerous lesions. J. Natl. Cancer Inst..

[11] González C.A., Figueiredo C., Lic C.B., Ferreira R.M., Pardo M.L., Ruiz Liso J.M., Alonso P., Sala N., Capella G., Sanz-Anquela J.M. (2011). *Helicobacter pylori* cagA and vacA genotypes as predictors of progression of gastric preneoplastic lesions: A long-term follow-up in a high-risk area in Spain. Am. J. Gastroenterol..

[12] Figura N., Marano L., Moretti E., Ponzetto A. (2016). *Helicobacter pylori* infection and gastric carcinoma: Not all the strains and patients are alike. World J. Gastrointest. Oncol..

[13] Figura N., Valassina M., Moretti E., Vindigni C., Collodel G., Iacoponi F., Giordano N., Roviello F., Marrelli D. (2015). Histological variety of gastric carcinoma and *Helicobacter pylori* cagA and vacA polymorphism. Eur. J. Gastroenterol. Hepatol..

[14] Repetto O., Zanussi S., Casarotto M., Canzonieri V., De Paoli P., Cannizzaro R., De Re V. (2014). Differential proteomics of *Helicobacter pylori* associated with autoimmune atrophic gastritis. Mol. Med..

[15] Bernardini G., Figura N., Ponzetto A., Marzocchi B., Santucci A. (2017). Application of proteomics to the study of *Helicobacter pylori* and implications for the clinic. Expert Rev. Proteom..

[16] Karita M., Blaser M.J. (1998). Acid-tolerance response in *Helicobacter pylori* and differences between cagA+ and cagA- strains. J. Infect. Dis..

[17] Suerbaum S., Michetti P. (2002). *Helicobacter pylori* infection. N. Engl. J. Med..

[18] Figura N., Trabalzini L., Mini R., Bernardini G., Scaloni A., Talamo F., Lusini P., Ferro E., Martelli P., Santucci A. (2004). Inactivation of *Helicobacter pylori* cagA gene affects motility. Helicobacter.

[19] Basaglia G., Sperandio P., Tomasini M.L., Calzavara S.S., Giordari F., De Paoli P. (2004). Analysis of antimicrobial susceptibility and virulence factors in *Helicobacter pylori* clinical isolates. J. Chemother..

[20] De Paoli P., Tomasini M.L., Basaglia G. (2004). The predictive value of *Helicobacter pylori* in-vitro metronidazole resistance. Clin. Microbiol. Infect..

[21] Tomasini M.L., Zanussi S., Sozzi M., Tedeschi R., Basaglia G., De Paoli P. (2003). Heterogeneity of cag genotypes in *Helicobacter pylori* isolates from human biopsy specimens. J. Clin. Microbiol..

[22] Sozzi M., Crosatti M., Kim S.K., Romero J., Blaser M.J. (2001). Heterogeneity of *Helicobacter pylori* cag genotypes in experimentally infected mice. FEMS Microbiol. Lett..

[23] Sozzi M., Valentini M., Figura N., De Paoli P., Tedeschi R.M., Gloghini A., Serraino D., Poletti M., Carbone A. (1998). Atrophic gastritis and intestinal metaplasia in *Helicobacter pylori* infection: The role of CagA status. Am. J. Gastroenterol..

[24] Sozzi M., Tomasini M.L., Vindigni C., Zanussi S., Tedeschi R., Basaglia G., Figura N., De Paoli P. (2005). Heterogeneity of cag genotypes and clinical outcome of *Helicobacter pylori* infection. J. Lab. Clin. Med..

[25] Wroblewski L.E., Peek R.M., Wilson K.T. (2010). *Helicobacter pylori* and gastric cancer: Factors that modulate disease risk. Clin. Microbiol. Rev..

[26] Jang S., Jones K.R., Olsen C.H., Joo Y.M., Yoo Y.J., Chung I.S., Cha J.H., Merrell D.S. (2010). Epidemiological link between gastric disease and polymorphisms in VacA and CagA. J. Clin. Microbiol..

[27] Cover T.L., Tummuru M.K., Cao P., Thompson S.A., Blaser M.J. (1994). Divergence of genetic sequences for the vacuolating cytotoxin among *Helicobacter pylori* strains. J. Biol. Chem..

[28] Rhead J.L., Letley D.P., Mohammadi M., Hussein N., Mohagheghi M.A., Eshagh Hosseini M., Atherton J.C. (2007). A new *Helicobacter pylori* vacuolating cytotoxin determinant, the intermediate region, is associated with gastric cancer. Gastroenterology.

[29] Basso D., Zambon C.F., Letley D.P., Stranges A., Marchet A., Rhead J.L., Schiavon S., Guariso G., Ceroti M., Nitti D. (2008). Clinical relevance of *Helicobacter pylori* cagA and vacA gene polymorphisms. Gastroenterology.

[30] Fahimi F., Tohidkia M.R., Fouladi M., Aghabeygi R., Samadi N., Omidi Y. (2017). Pleiotropic cytotoxicity of VacA toxin in host cells and its impact on immunotherapy. Bioimpacts.

[31] Oleastro M., Cordeiro R., Ferrand J., Nunes B., Lehours P., Carvalho-Oliveira I., Mendes A.I., Penque D., Monteiro L., Mégraud F. (2008). Evaluation of the clinical significance of homB, a novel candidate marker of *Helicobacter pylori* strains associated with peptic ulcer disease. J. Infect. Dis..

[32] Jung S.W., Sugimoto M., Graham D.Y., Yamaoka Y. (2009). homB status of *Helicobacter pylori* as a novel marker to distinguish gastric cancer from duodenal ulcer. J. Clin. Microbiol..

[33] Talebi Bezmin Abadi A., Rafiei A., Ajami A., Hosseini V., Taghvaei T., Jones K.R., Merrell D.S. (2011). *Helicobacter pylori* homB, but not cagA, is associated with gastric cancer in Iran. J. Clin. Microbiol..

[34] Oleastro M., Monteiro L., Lehours P., Mégraud F., Ménard A. (2006). Identification of markers for *Helicobacter pylori* strains isolated from children with peptic ulcer disease by suppressive subtractive hybridization. Infect. Immun..

[35] Atherton J.C. (2006). The pathogenesis of *Helicobacter pylori*-induced gastro-duodenal diseases. Annu. Rev. Pathol..

[36] Mommersteeg M.C., Yu J., Peppelenbosch M.P., Fuhler G.M. (2018). Genetic host factors in *Helicobacter pylori*-induced carcinogenesis: Emerging new paradigms. Biochim. Biophys. Acta.

[37] Liu H., Fero J.B., Mendez M., Carpenter B.M., Servetas S.L., Rahman A., Goldman M.D., Boren T., Salama N.R., Merrell D.S. (2015). Analysis of a single *Helicobacter pylori* strain over a 10-year period in a primate model. Int. J. Med. Microbiol..

[38] Thompson L.J., Danon S.J., Wilson J.E., O’Rourke J.L., Salama N.R., Falkow S., Mitchell H., Lee A. (2004). Chronic *Helicobacter pylori* infection with Sydney strain 1 and a newly identified mouse-adapted strain (Sydney strain 2000) in C57BL/6 and BALB/c mice. Infect. Immun..

[39] Brenner H., Arndt V., Stürmer T., Stegmaier C., Ziegler H., Dhom G. (2000). Individual and joint contribution of family history and *Helicobacter pylori* infection to the risk of gastric carcinoma. Cancer.

[40] Toh B.H. (2014). Diagnosis and classification of autoimmune gastritis. Autoimmun. Rev..

[41] Yaghoobi M., Bijarchi R., Narod S.A. (2010). Family history and the risk of gastric cancer. Br. J. Cancer.

[42] Stec-Michalska K., Peczek L., Michalski B., Wisniewska-Jarosinska M., Krakowiak A., Nawrot B. (2009). *Helicobacter pylori* infection and family history of gastric cancer decrease expression of FHIT tumor suppressor gene in gastric mucosa of dyspeptic patients. Helicobacter.

[43] Siavoshi F., Asgharzadeh A., Ghadiri H., Massarrat S., Latifi-Navid S., Zamani M. (2011). *Helicobacter pylori* genotypes and types of gastritis in first-degree relatives of gastric cancer patients. Int. J. Med. Microbiol..

[44] Queiroz D.M., Silva C.I., Goncalves M.H., Braga-Neto M.B., Fialho A.B., Fialho A.M., Rocha G.A., Rocha A.M., Batista S.A., Guerrant R.L. (2012). Higher frequency of cagA EPIYA-C phosphorylation sites in *H. pylori* strains from first-degree relatives of gastric cancer patients. BMC Gastroenterol..

[45] Marcos-Pinto R., Dinis-Ribeiro M., Carneiro F., Wen X., Lopes C., Figueiredo C., Machado J.C., Ferreira R.M., Reis C.A., Canedo P. (2013). First-degree relatives of early-onset gastric cancer patients show a high risk for gastric cancer: Phenotype and genotype profile. Virchows Arch..

[46] Venerito M., Radünz M., Reschke K., Reinhold D., Frauenschläger K., Jechorek D., Di Mario F., Malfertheiner P. (2015). Autoimmune gastritis in autoimmune thyroid disease. Aliment. Pharmacol. Ther..

[47] Zhang Y., Weck M.N., Schöttker B., Rothenbacher D., Brenner H. (2013). Gastric parietal cell antibodies, *Helicobacter pylori* infection, and chronic atrophic gastritis: Evidence from a large population-based study in Germany. Cancer Epidemiol. Biomark. Prev..

[48] Yadegar A., Alebouyeh M., Zali M.R. (2015). Analysis of the intactness of *Helicobacter pylori* cag pathogenicity island in Iranian strains by a new PCR-based strategy and its relationship with virulence genotypes and EPIYA motifs. Infect. Genet. Evol..

[49] Khatoon J., Prasad K.N., Prakash Rai R., Ghoshal U.C., Krishnani N. (2017). Association of heterogenicity of *Helicobacter pylori* cag pathogenicity island with peptic ulcer diseases and gastric cancer. Br. J. Biomed. Sci..

[50] Achtman M., Azuma T., Berg D.E., Ito Y., Morelli G., Pan Z.J., Suerbaum S., Thompson S.A., van der Ende A., van Doorn L.J. (1999). Recombination and clonal groupings within *Helicobacter pylori* from different geographical regions. Mol. Microbiol..

[51] Parsonnet J., Friedman G.D., Orentreich N., Vogelman H. (1997). Risk for gastric cancer in people with CagA positive or CagA negative *Helicobacter pylori* infection. Gut.

[52] Queiroz D.M., Mendes E.N., Rocha G.A., Oliveira A.M., Oliveira C.A., Magalhães P.P., Moura S.B., Cabral M.M., Nogueira A.M. (1998). cagA-positive *Helicobacter pylori* and risk for developing gastric carcinoma in Brazil. Int. J. Cancer.

[53] Torres J., Pérez-Pérez G.I., Leal-Herrera Y., Muñoz O. (1998). Infection with CagA+ *Helicobacter pylori* strains as a possible predictor of risk in the development of gastric adenocarcinoma in Mexico. Int. J. Cancer.

[54] Miehlke S., Kirsch C., Agha-Amiri K., Günther T., Lehn N., Malfertheiner P., Stolte M., Ehninger G., Bayerdörffer E. (2000). The *Helicobacter pylori* vacA s1, m1 genotype and cagA is associated with gastric carcinoma in Germany. Int. J. Cancer.

[55] Sheikh A.F., Yadyad M.J., Goodarzi H., Hashemi S.J., Aslani S., Assarzadegan M.A., Ranjbar R. (2018). CagA and vacA allelic combination of *Helicobacter pylori* in gastroduodenal disorders. Microb. Pathog..

[56] Kang J., Jones K.R., Jang S., Olsen C.H., Yoo Y.J., Merrell D.S., Cha J.H. (2012). The geographic origin of *Helicobacter pylori* influences the association of the homB gene with gastric cancer. J. Clin. Microbiol..

[57] Kauser F., Khan A.A., Hussain M.A., Carroll I.M., Ahmad N., Tiwari S., Shouche Y., Das B., Alam M., Ali S.M. (2004). The cag pathogenicity island of *Helicobacter pylori* is disrupted in the majority of patient isolates from different human populations. J. Clin. Microbiol..

[58] Kennemann L., Didelot X., Aebischer T., Kuhn S., Drescher B., Droege M., Reinhardt R., Correa P., Meyer T.F., Josenhans C. (2011). *Helicobacter pylori* genome evolution during human infection. Proc. Natl. Acad. Sci. USA.

[59] Motta C.R., Cunha M.P., Queiroz D.M., Cruz F.W., Guerra E.J., Mota R.M., Braga L.L. (2008). Gastric precancerous lesions and *Helicobacter pylori* infection in relatives of gastric cancer patients from Northeastern Brazil. Digestion.

[60] Rokkas T., Sechopoulos P., Pistiolas D., Margantinis G., Koukoulis G. (2010). *Helicobacter pylori* infection and gastric histology in first-degree relatives of gastric cancer patients: A meta-analysis. Eur. J. Gastroenterol. Hepatol..

[61] Liao J., Wen S., Cao L., Zhou Y., Feng Z. (2015). Effect of eradication of *Helicobacter pylori* on expression levels of FHIT, IL-8 and P73 in gastric mucosa of first-degree relatives of gastric cancer patients. PLoS ONE.

[62] Vilkin A., Levi Z., Morgenstern S., Shmuely H., Gal E., Hadad B., Hardi B., Niv Y. (2008). Higher gastric mucin secretion and lower gastric acid output in first-degree relatives of gastric cancer patients. J. Clin. Gastroenterol..

[63] Murphy G., Dawsey S.M., Engels E.A., Ricker W., Parsons R., Etemadi A., Lin S.W., Abnet C.C., Freedman N.D. (2015). Cancer risk after pernicious anemia in the US elderly population. Clin. Gastroenterol. Hepatol..

[64] Mahmud N., Stashek K., Katona B.W., Tondon R., Shroff S.G., Roses R., Furth E.E., Metz D.C. (2019). The incidence of neoplasia in patients with autoimmune metaplastic atrophic gastritis: A renewed call for surveillance. Ann. Gastroenterol..

[65] Annibale B., Lahner E., Negrini R., Baccini F., Bordi C., Monarca B., Delle Fave G. (2005). Lack of specific association between gastric autoimmunity hallmarks and clinical presentations of atrophic body gastritis. World J. Gastroenterol..

[66] Rugge M., Fassan M., Pizzi M., Zorzetto V., Maddalo G., Realdon S., De Bernard M., Betterle C., Cappellesso R., Pennelli G. (2012). Autoimmune gastritis: Histology phenotype and OLGA staging. Aliment. Pharmacol. Ther..

[67] Presotto F., Sabini B., Cecchetto A., Plebani M., De Lazzari F., Pedini B., Betterle C. (2003). *Helicobacter pylori* infection and gastric autoimmune diseases: Is there a link?. Helicobacter.

[68] Minalyan A., Benhammou J.N., Artashesyan A., Lewis M.S., Pisegna J.R. (2017). Autoimmune atrophic gastritis: Current perspectives. Clin. Exp. Gastroenterol..

[69] Parsons B.N., Ijaz U.Z., D’Amore R., Burkitt M.D., Eccles R., Lenzi L., Duckworth C.A., Moore A.R., Tiszlavicz L., Varro A. (2017). Comparison of the human gastric microbiota in hypochlorhydric states arising as a result of *Helicobacter pylori*-induced atrophic gastritis, autoimmune atrophic gastritis and proton pump inhibitor use. PLoS Pathog..

[70] Klymiuk I., Bilgilier C., Stadlmann A., Thannesberger J., Kastner M.T., Högenauer C., Püspök A., Biowski-Frotz S., Schrutka-Kölbl C., Thallinger G.G. (2017). The human gastric microbiome is predicated upon infection with *Helicobacter pylori*. Front. Microbiol..

[71] Adamsson I., Edlund C., Nord C.E. (2000). Impact of treatment of *Helicobacter pylori* on the normal gastrointestinal microflora. Clin. Microbiol. Infect..

[72] Jakobsson H., Wreiber K., Fall K., Fjelstad B., Nyrén O., Engstrand L. (2007). Macrolide resistance in the normal microbiota after *Helicobacter pylori* treatment. Scand. J. Infect. Dis..

[73] Zanussi S., Casarotto M., Basaglia G., Tedeschi R., Giacomini S., Canzonieri V., De Re V., Maiero S., Cannizzaro R., De Paoli P. (2013). Prevalence of *Helicobacter pylori* infection and its genetic heterogeneity in autoimmune atrophic chronic gastritis patients. Helicobacter.

[74] Kraft C., Stack A., Josenhans C., Niehus E., Dietrich G., Correa P., Fox J.G., Falush D., Suerbaum S. (2006). Genomic changes during chronic *Helicobacter pylori* infection. J. Bacteriol..

[75] Lan R., Reeves P.R. (2000). Intraspecies variation in bacterial genomes: The need for a species genome concept. Trends Microbiol..

[76] D’Elios M.M., Appelmelk B.J., Amedei A., Bergman M.P., Del Prete G. (2004). Gastric autoimmunity: The role of *Helicobacter pylori* and molecular mimicry. Trends Mol. Med..

[77] Amedei A., Bergman M.P., Appelmelk B.J., Azzurri A., Benagiano M., Tamburini C., van der Zee R., Telford J.L., Vandenbroucke-Grauls C.M., D’Elios M.M. (2003). Molecular mimicry between *Helicobacter pylori* antigens and H+, K+ --adenosine triphosphatase in human gastric autoimmunity. J. Exp. Med..

[78] Roujeinikova A. (2014). Phospholipid binding residues of eukaryotic membrane-remodelling F-BAR domain proteins are conserved in *Helicobacter pylori* CagA. BMC Res. Notes.

[79] Kalim K.W., Yang J.Q., Li Y., Meng Y., Zheng Y., Guo F. (2018). Reciprocal regulation of glycolysis-driven Th17 pathogenicity and regulatory T cell stability by Cdc42. J. Immunol..

[80] Chmiela M., Gonciarz W. (2017). Molecular mimicry in *Helicobacter pylori* infections. World J. Gastroenterol..

[81] Roy A., Ganesh G., Sippola H., Bolin S., Sawesi O., Dagälv A., Schlenner S.M., Feyerabend T., Rodewald H.R., Kjellén L. (2014). Mast cell chymase degrades the alarmins heat shock protein 70, biglycan, HMGB1, and interleukin-33 (IL-33) and limits danger-induced inflammation. J. Biol. Chem..

[82] Lennon E.M., Borst L.B., Edwards L.L., Moeser A.J. (2018). Mast cells exert anti-inflammatory effects in an IL10-/- model of spontaneous colitis. Mediators Inflamm..

[83] Zárate-Bladés C.R., Horai R., Caspi R.R. (2016). Regulation of autoimmunity by the Microbiome. DNA Cell Biol..

[84] Augustyniak D., Majkowska-Skrobek G., Roszkowiak J., Dorotkiewicz-Jach A. (2017). Defensive and offensive cross-reactive antibodies elicited by pathogens: The good, the bad and the ugly. Curr. Med. Chem..

[85] Gressmann H., Linz B., Ghai R., Pleissner K.P., Schlapbach R., Yamaoka Y., Kraft C., Suerbaum S., Meyer T.F., Achtman M. (2005). Gain and loss of multiple genes during the evolution of *Helicobacter pylori*. PLoS Genet..

[86] Oh J.D., Kling-Bäckhed H., Giannakis M., Xu J., Fulton R.S., Fulton L.A., Cordum H.S., Wang C., Elliott G., Edwards J. (2006). The complete genome sequence of a chronic atrophic gastritis *Helicobacter pylori* strain: Evolution during disease progression. Proc. Natl. Acad. Sci. USA.

[87] Suzuki R., Shiota S., Yamaoka Y. (2012). Molecular epidemiology, population genetics, and pathogenic role of *Helicobacter pylori*. Infect. Genet. Evol..

[88] Vilaichone R.K., Mahacahai V., Tumwasorn S., Kachintorn U. (2011). CagA genotype and metronidazole resistant strain of *Helicobacter pylori* in functional dyspepsia in Thailand. J. Gastroenterol. Hepatol..

[89] Bachir M., Allem R., Tifrit A., Medjekane M., Drici A.E., Diaf M., Douidi K.T. (2018). Primary antibiotic resistance and its relationship with cagA and vacA genes in *Helicobacter pylori* isolates from Algerian patients. Braz. J. Microbiol..

[90] Fasciana T., Calà C., Bonura C., Di Carlo E., Matranga D., Scarpulla G., Manganaro M., Camilleri S., Giammanco A. (2015). Resistance to clarithromycin and genotypes in *Helicobacter pylori* strains isolated in Sicily. J. Med. Microbiol..

[91] Brennan D.E., Dowd C., O’Morain C., McNamara D., Smith S.M. (2018). Can bacterial virulence factors predict antibiotic resistant *Helicobacter pylori* infection?. World J. Gastroenterol..

[92] Sugimoto M., Yamaoka Y. (2009). Virulence factor genotypes of *Helicobacter pylori* affect cure rates of eradication therapy. Arch. Immunol. Ther. Exp. (Warsz.).

[93] Wong B.C., Wang W.H., Berg D.E., Fung F.M., Wong K.W., Wong W.M., Lai K.C., Cho C.H., Hui W.M., Lam S.K. (2001). High prevalence of mixed infections by *Helicobacter pylori* in Hong Kong: Metronidazole sensitivity and overall genotype. Aliment. Pharmacol Ther..

[94] Ben Mansour K., Fendri C., Battikh H., Garnier M., Zribi M., Jlizi A., Burucoa C. (2016). Multiple and mixed *Helicobacter pylori* infections: Comparison of two epidemiological situations in Tunisia and France. Infect. Genet. Evol..

[95] Matteo M.J., Armitano R.I., Granados G., Wonaga A.D., Sánches C., Olmos M., Catalano M. (2010). *Helicobacter pylori* oipA, vacA and dupA genetic diversity in individual hosts. J. Med. Microbiol..

[96] Zhang Y., Wang S., Hu B., Zhao F., Xiang P., Ji D., Chen F., Liu X., Yang F., Wu Y. (2016). Direct detection of *Helicobacter pylori* in biopsy specimens using a high-throughput multiple genetic detection system. Future Microbiol..

[97] Isenberg H.D. (2004). Clinical Microbiology Procedure Handbook.

[98] Genta R.M. (1996). Recognizing atrophy: Another step toward a classification of gastritis. Am. J. Surg. Pathol..

[99] Schmidt H.M., Andres S., Nilsson C., Kovach Z., Kaakoush N.O., Engstrand L., Goh K.L., Fock K.M., Forman D., Mitchell H. (2010). The cag PAI is intact and functional but HP0521 varies significantly in *Helicobacter pylori* isolates from Malaysia and Singapore. Eur. J. Clin. Microbiol. Infect. Dis..

[100] Chattopadhyay S., Patra R., Ramamurthy T., Chowdhury A., Santra A., Dhali G.K., Bhattacharya S.K., Berg D.E., Nair G.B., Mukhopadhyay A.K. (2004). Multiplex PCR assay for rapid detection and genotyping of *Helicobacter pylori* directly from biopsy specimens. J. Clin. Microbiol..

[101] Lash J.G., Genta R.M. (2013). Adherence to the Sydney System guidelines increases the detection of *Helicobacter* gastritis and intestinal metaplasia in 400738 sets of gastric biopsies. Aliment. Pharmacol. Ther..

[102] Malfertheiner P., Megraud F., O’Morain C.A., Gisbert J.P., Kuipers E.J., Axon A.T., Bazzoli F., Gasbarrini A., Atherton J., Graham D.Y. (2017). European helicobacter and microbiota study group and consensus panel. Management of *Helicobacter pylori* infection-the Maastricht V/Florence Consensus Report. Gut.

[103] Espinoza J.L., Matsumoto A., Tanaka H., Matsumura I. (2018). Gastric microbiota: An emerging player in *Helicobacter pylori*-induced gastric malignancies. Cancer Lett..

[104] Meng C., Bai C., Brown T.D., Hood L.E., Tian Q. (2018). Human gut microbiota and gastrointestinal cancer. Genom. Proteom. Bioinform..

