# Supplementary material for: Differential Helicobacter pylori Plasticity in the Gastric Niche of Subjects at Increased Gastric Cancer Risk
Source: Pathogens. 2019 May 18;8(2):65. doi: 10.3390/pathogens8020065 (PMC6630233; doi:10.3390/pathogens8020065)
Supplement: Supplementary file 1 [file pathogens-08-00065-s001.pdf]

Supplementary Table S1

Sydney classification of histological parameters in AG patients by gastric topography

	AG (N=14)		
Variable	Corpus	Antrum	p-value*
Atrophy ≥1, n. (%)	9 (64.29)	11 (78.57)	0.68
Intestinal metaplasia ≥1, n. (%)	1(7.14)	3 (21.43)	0.60
Activity ≥1, n. (%)	9 (64.29)	10 (71.43)	1.00
Inflammation 2-3, n. (%)	4 (28.57)	6 (42.86)	0.70
HP density 2-3, n. (%)	1 (7.14)	1 (7.14)	1.00

AG, Autoimmune Gastritis; *, p-value by Fisher exact test; HP, *Helicobacter pylori*.
